# Supplementary material for: Governing antibiotic resistance through One Health: Insights from the political and legal landscape in Senegal
Source: PLOS Glob Public Health. 2026 Mar 12;6(3):e0005889. doi: 10.1371/journal.pgph.0005889 (PMC12981456; doi:10.1371/journal.pgph.0005889)
Supplement: S1 Table — (DOCX) [file pgph.0005889.s001.docx]

S1 Table. Regional and national institutions involved in antibiotic resistance management and their main legal instruments

| Name | Sector | Professional category | Mission | Instruments |
| --- | --- | --- | --- | --- |
| African Union Center for Disease Control and Prevention | Multi-sectoral | Scientific and technical institute | Support of Member States' public health initiatives and strengthen of the capacity of their public health institutions | Africa's common position on antimicrobial resistance  African regional antimicrobial resistance communication and awareness strategy  African Center for Disease Control and Prevention framework for antimicrobial resistance 2018-2023 |
| West African Economic and Monetary Union | Multi-sectoral | Regional economic organization | Harmonization of the legal environment among member countries | Regulation no. 01/2006/CM/West African Economic and Monetary Union of March 23, 2006 establishing and operating modalities of the Veterinary Committee within West African Economic and Monetary Union  Decision no. 09/2010/CM/West African Economic and Monetary Union of October 1, 2010 adopting the guide to good practices for the distribution and importation of pharmaceutical products for human use in the member states of the West African Economic and Monetary Union  Directive no. 07/2006/CM/West African Economic and Monetary Union relating to veterinary pharmacy |
| National One Health platform | Multi-sectoral | Governmental authorities | Multi-sectoral coordination of multi-sectoral health policy development, implementation and evaluation | National multisectoral action plan for combating antimicrobial resistance (2024-2028)  National antimicrobial stewardship plan |
| National committee of Codex Alimentarius | Agriculture and food | Scientific and technical institute | Monitoring implementation of food standards to protect consumers’ health and ensure fair trade practices | Order no. 12 May 2017 - 07951 establishing the monitoring plan for veterinary drug residues, chemical, biological and microbiological contaminants in aquaculture products in Senegal |
| National Food Analysis and Control Laboratory | Agriculture and food | Scientific and technical institute | Control of the quality of food and non-food products | Law no. 2014-21 of May 7, 2014 creating a public industrial and commercial establishment called the National Analysis and Control Laboratory |
| Directorate of Veterinary Services | Animal health | Governmental authorities | Development and implementation of policies and regulations on veterinary antimicrobial use and stewardship | Law no. 66-48 of May 27, 1966 relating to the control of food products and the repression of fraud  Law no. 2008-07 of January 24, 2008 organizing the veterinary profession and pharmacy |
| Veterinary council of Senegal | Animal health | Professional organizations | Development and enforcement of the professional ethic code for veterinary practice | Law no. 92-52 of July 10, 1992 establishing the Code for the creation of the Order of Veterinary Doctors of Senegal  Decree no. 93-514 of April 27, 1993 relating to the Code of Ethics |
| Directorate of Sanitation | Environment | Governmental authorities | Development and implementation of policies regulations on wastewater | Law no. 2009-24 of July 8, 2009 relating to the Sanitation Code |
| Directorate of the Environment and Classified Establishments | Environment | Governmental authorities | Development and implementation of policies regulations on effluents | Law no. 2023-15 of August 2, 2023 relating to the Environmental Code  Law no. 2022-20 of June 14, 2022 relating to Biosecurity |
| Directorate of Laboratories | Human health | Governmental authorities | Development and implementation of policies regulations on laboratory activities | Law no. 75-409 of May 29, 1975 relating to the Health Code  Integrated surveillance manual for antimicrobial resistance in Senegal |
| Senegalese pharmaceutical regulatory agency | Human health | Governmental authorities | Development, implementation and monitoring of policies and regulations on human health antimicrobial use and stewardship | Law no. 54-418 of April 15, 1954 extending certain provisions of the Public Health Code to the Overseas Territories, Togo and Cameroon  Law no. 2023-06 of June 13, 2023 relating to medicines, other health products and pharmacies of Monday June 5, 2023 |
| Pharmacist council of Senegal | Human health | Professional organizations | Development and enforcement of the professional ethic code | Law no. 73-62 of December 19, 1973 creating the Order of Pharmacists of Senegal |
