# Supplementary material for: Governing antibiotic resistance through One Health: Insights from the political and legal landscape in Senegal
Source: PLOS Glob Public Health. 2026 Mar 12;6(3):e0005889. doi: 10.1371/journal.pgph.0005889 (PMC12981456; doi:10.1371/journal.pgph.0005889)
Supplement: S2 Table — (DOCX) [file pgph.0005889.s002.docx]

S2 Table. Surveillance programmes for antibiotic resistance, use, quality and residues in Senegal

| Name | Year | Sector | Owner | Object | Population  Commodities | Collection points | Surveillance strategy |
| --- | --- | --- | --- | --- | --- | --- | --- |
| Surveillance system for antibiotic residues in agricultural products | 1963 | Agriculture and food | Ministry of Industry | Residues | Foodstuffs | Food processing units | Event-based surveillance |
| Surveillance system for antibiotic residues in foodstuffs | 2014 | Agriculture and food | Ministry of Commerce | Residues | Foodstuffs | Food retail units | Event-based surveillance |
| Surveillance system for resistant bacteria in animal | 2005 | Animal health | Ministry of Livestock | Resistances | Food producing animals  Companion animals | Veterinary practice | Event-based surveillance |
| Surveillance system for imports of veterinary drugs | 2009 | Animal health | Ministry of Livestock | Use | Food producing animals  Companion animals | Drug importation compagnies | Event-based surveillance |
| National Health Information System | 2016 | Human health | Ministry of Health | Resistance | Patient | Medical practice | Event-based surveillance |
| Surveillance system for imports of human drugs | 2022 | Human health | Ministry of Health | Use | Users | Drug importation compagnies | Event-based surveillance |
| Surveillance system for the distribution and consumption of drugs | 1954 | Human health | Ministry of Health | Use | Patient | Hospital and retail pharmacies | Event-based surveillance |
| Pharmacovigilance system | 1998 | Human health | Ministry of Health | Quality | Users | Medical practice | Event-based surveillance |
