# Supplementary material for: Governing antibiotic resistance through One Health: Insights from the political and legal landscape in Senegal
Source: PLOS Glob Public Health. 2026 Mar 12;6(3):e0005889. doi: 10.1371/journal.pgph.0005889 (PMC12981456; doi:10.1371/journal.pgph.0005889)
Supplement: Attachment — Submitted filename: PGPH-D-25-03107_reviewer.pdf [file pgph.0005889.s003.pdf]

PLOS Global Public Health

One Health governance of antibiotic resistance in Senegal

--Manuscript Draft--

**Manuscript Number:**	PGPH-D-25-03107
**Article Type:**	Research Article
**Full Title:**	One Health governance of antibiotic resistance in Senegal
**Short Title:**	One Health governance of antibiotic resistance in Senegal
**Corresponding Author:**	Marion Bordier<br>CIRAD<br>Dakar, SENEGAL
**Order of Authors:**	Mouhamadou Moustapha Sow, PhD Student
	Léo Delpy
	Mamadou Ciss
	Assane Gueye Fall
	Nicolas Djighnoum Diouf
	Jean-Hugues Caffin
	Ndeye Mery Dia
	Marion Bordier
**Keywords:**	Antibiotic Resistance; governance; One Health; Senegal
**Abstract:**	Antimicrobial resistance (AMR), especially antibiotic resistance (ABR), could cause up to 10 million deaths by 2050. While ABR is a natural phenomenon, it is exacerbated by antibiotic misuse, poor sanitation, insufficient health infrastructure, weak biosecurity, and environmental contamination. The cross transmission of resistant bacteria between human-animal-environment requires multi-sectoral response inspired by the One Health concept. With the support of international partners and in line with global policies, Senegal has established a national One Health platform and legal measures to combat ABR. Our study proposes to evaluate the political and institutional framework for managing ABR in Senegal, from a One Health perspective.<br>A qualitative approach was adopted, combining the review of 64 legal instruments and 26 semi-structured interviews conducted between January and August 2023 with actors form governmental, private and non-governmental institutions. The analysis focused on the governance structure, legal instrument development and implementation, and ABR surveillance programmes. The thematic analysis of the interview contents highlighted factors influencing the One Health governance capacities in Senegal.<br>One Health governance has been institutionalized in Senegal since 2017 with the creation of the national One Health platform housed at the Prime Minister's Office. However, its effectiveness is limited by weak formalization, inadequate resources, and reliance on external funding. Outdated and poorly enforced legal frameworks hinder ABR management, while rural areas lack sufficient laboratory infrastructure, leading to inappropriate antibiotic use and weak surveillance systems. Power imbalances among stakeholders impede collective action and data-sharing.<br>Despite progress toward international standards, systemic barriers persist. Strengthening the legal framework, improving healthcare infrastructure, and enhancing the One Health platform’s status and funding are critical. Institutionalizing must bridge global orientations with local realities to avoid inequities. Sustainable ABR management requires inclusive, context-sensitive policies, robust domestic investment, and equitable collaboration among stakeholders across the sectors of human, animal, and environmental health.
**Additional Information:**	
**Question**	**Response**

Powered by Editorial Manager® and ProduXion Manager® from Aries Systems Corporation

**Financial Disclosure**<br><br>Enter a financial disclosure statement that describes the sources of funding for the work included in this submission and the role the funder(s) played. This includes grants and any commercial funding of the work or authors.<br><br>This statement will be typeset if the manuscript is accepted for publication.<br><br>*Please review the submission guidelines and the instructions link below for detailed requirements and guidance.*	This study is part of the Designing One Health Governance for Antimicrobial Stewardship Interventions (DESIGN) project. It was funded by the Swedish International Development Cooperation Agency (SIDA) and the French National Research Agency (Agence Nationale de la Recherche, or ANR), through the Joint Programming Initiative on Antimicrobial Resistance (JPI-AMR)
**Competing Interests**<br><br>On behalf of all authors, disclose any competing interests that could be perceived to bias this work.<br><br>This statement will be typeset if the manuscript is accepted for publication.<br><br>*Please review the instructions link below and PLOS Global Public Health's competing interests policy to determine what information must be disclosed at submission.*	The authors declare that they have no competing interests.
**Data Availability**<br><br>Before publication, Authors are required to make fully available and without restriction all data underlying their findings. Please see our PLOS Data Policy page for detailed information on this policy.<br><br>A **Data Availability Statement**, detailing where the data can be accessed, is required at first submission. Insert your	The full dataset generated during the current study is available from the corresponding author on reasonable request.

Powered by Editorial Manager® and ProduXion Manager® from Aries Systems Corporation

Data Availability Statement in the box below.<br><br>Please see the data reporting section of our submission guidelines for instructions on what you need to include in your Data Availability Statement.<br><br>This statement will be typeset if the manuscript is accepted for publication.<br><br>*PLOS allows rare exemptions to address legal and ethical concerns. If you have legal or ethical restrictions, please use the box below to detail these in full sentences for the Journal team to consider.*	

Manuscript

Click here to access/download;Manuscript;20250902_Capacity One Health Governance_ PGPH.docx

One Health governance of antibiotic resistance in Senegal

Mouhamadou Moustapha Sow[1,2], Léo Delpy[3,4], Mamadou Ciss[2], Assane Gueye Fall[2], Nicolas Djighnoum Diouf[5], Jean-Hugues Caffin[4,6], Ndeye Mery Dia[1] and Marion Bordier[2,4*]

[1]Health Science and Research Laboratory, Gaston Berger University, Saint Louis, Senegal

[2]National Laboratory for Livestock and Veterinary research, Senegalese Institute of Agricultural Research, Dakar, Senegal

[3]UMR 8019, University of Lille, Villeneuve d'Ascq, France

[4]ASTRE, University of Montpellier, CIRAD, INRAE, Montpellier, France

[5]Laboratory of Biological, Agronomic, Food Sciences and Modelling of Complex Systems, Gaston Berger University, Saint Louis, Senegal

[6]UMR ASTRE, University of Montpellier, CIRAD, INRAE, Montpellier, France

[7]CIRAD, UMR ASTRE, Dakar, Senegal

* corresponding author

Email: marion.bordier@cirad.fr

Abstract

Antimicrobial resistance (AMR), especially antibiotic resistance (ABR), could cause up to 10 million deaths by 2050. While ABR is a natural phenomenon, it is exacerbated by antibiotic misuse, poor sanitation, insufficient health infrastructure, weak biosecurity, and environmental contamination. The cross transmission of resistant bacteria between human-animal-environment requires multi-sectoral response inspired by the One Health concept. With the support of international partners and in line with global policies, Senegal has established a

national One Health platform and legal measures to combat ABR. Our study proposes to evaluate the political and institutional framework for managing ABR in Senegal, from a One Health perspective.

A qualitative approach was adopted, combining the review of 64 legal instruments and 26 semi-structured interviews conducted between January and August 2023 with actors form governmental, private and non-governmental institutions. The analysis focused on the governance structure, legal instrument development and implementation, and ABR surveillance programmes. The thematic analysis of the interview contents highlighted factors influencing the One Health governance capacities in Senegal.

One Health governance has been institutionalized in Senegal since 2017 with the creation of the national One Health platform housed at the Prime Minister's Office. However, its effectiveness is limited by weak formalization, inadequate resources, and reliance on external funding. Outdated and poorly enforced legal frameworks hinder ABR management, while rural areas lack sufficient laboratory infrastructure, leading to inappropriate antibiotic use and weak surveillance systems. Power imbalances among stakeholders impede collective action and data-sharing.

Despite progress toward international standards, systemic barriers persist. Strengthening the legal framework, improving healthcare infrastructure, and enhancing the One Health platform’s status and funding are critical. Institutionalizing must bridge global orientations with local realities to avoid inequities. Sustainable ABR management requires inclusive, context-sensitive policies, robust domestic investment, and equitable collaboration among stakeholders across the sectors of human, animal, and environmental health.

Introduction

Antimicrobial resistance (AMR) has become one of the greatest health issues of our time [1]. In 2015, initial estimates of the health impact of AMR pointed to 10 million deaths per year, with an estimated impact on GDP of $1 trillion per year by 2050 [2]. Antibiotic resistance (ABR) plays a key role in this major health problem. A recent study in 2022 directly attributes 1.27 million human deaths to bacterial resistance in 2019, with sub-Saharan Africa (SSA) accounting for the majority [3]. ABR thus could compromise efforts to achieve sustainable development goals in low middle income countries (LMICs) [4, 5].

ABR occurs when bacteria become resistant to antibiotics that were previously effective against them. It is the result of natural selection, exacerbated by other factors such as overuse, misuse and insufficient access to drinking water and sanitation [6, 7]. Resistant bacteria are found in the human, animal, and environmental sectors and can spread easily through direct contact, the food chain, and environmental pollution by effluents coming from livestock farms, hospitals and pharmaceutical facilities, and waste and sewage treatment plants [8 , 9 , 10 , 11 , 12]. Resistance is further amplified as bacteria exchange genetic material in shared ecosystems. Additionally, resistant bacteria and antibiotic residue can spread across geographical areas via trade and human travel [13 , 14]. Consequently, managing ABR requires a coordinated, multi-scale and multi-sectoral approach, in line with the One Health concept [15, 16, 17]. One Health is defined as an integrated, unifying approach that aims to sustainably balance and optimize the health of people, animals and ecosystems, and that recognizes the links between and interdependence of the health of humans, domestic and wild animals, plants, and the wider environment (including ecosystems) [18].

In response to the ABR crisis, the international community has committed to implementing effective and systemic policy measures to combat ABR, including the Global Action Plan on AMR, adopted by the World Health Assembly and issued by the World Health Organization (WHO) in 2015 [19], and recommendations on AMR developed by the Quadripartite, namely

the WHO, the Food and Agriculture Organization (FAO), the World Organization for Animal Health (WOAH), and the United Nations Environment Programme (UNEP) in 2024 [20]. Additionally, sectoral strategies have been issued by the FAO and the WOAH for the food and ~~the~~ animal ~~sector~~, respectively [21] [22]. These international plans provide guidance for countries to develop policy instruments and interventions, adapted to their national priorities and context, and promote effective multi-sectoral governance through the establishment of new politico-institutional arrangements to coordinate actions to combat ABR, in a One Health approach [23, 24]. In response to these international recommendations, Senegal has adapted its existing politico-institutional framework to better manage the ABR issue, through the establishment of a multi-sectoral institutional mechanism to coordinate actions of stakeholders involved in ABR management and the development of inter-sectoral legal instruments [25].

In this context, this study aims to examine the capacity of the current Senegalese politico-institutional framework to manage the ABR issue in a One Health perspective. Based on a review of the legal instruments related to ABR management and interviews with key stakeholders, we described the political and institutional landscape in relation to ABR management and identified determinants that could act as levers or barriers to effective One Health governance of ABR, and ways to improve this governance.

Materials and Methods

Governance is defined as the processes by which governmental and non-governmental stakeholders within civil society and the private sector exercise their power and authority and use their resources to influence, develop, manage and implement policies at local, national and global levels [26]. For this study, we have adapted this definition to include the One Health dimension applied to the ABR issue; we referred to One Health governance of ABR as the process by which stakeholders collaborate and coordinate their collective action to develop and

implement policies to manage the ABR issue, sometimes underpinned by an institutional and legal framework, and facilitated by effective management, and transparent mechanisms and relationships.

By ABR management, we refer to interventions that contribute to the prevention of the emergence and spread of ABR, through: (i) the reduction of the demand for antibiotics, including improving healthcare systems, biosecurity and hygiene practices; (ii) the judicious use of antibiotics, including reducing antibiotic misuse, controlling access to antibiotics, improving antibiotic quality; (iii) the surveillance of antibiotic use, resistance and residues.

We applied a qualitative approach, combining a document review and semi-structured interviews with stakeholders to: (i) describe the existing politico-institutional framework for ABR management in Senegal, i.e. institutions involved with ABR management and the related legal instruments they have issued and/or are implementing; (ii) identify drivers that may hinder or favour One Health governance of ABR management in Senegal; and (iii) describe potential ways of improving towards a more effective One Health governance.

Data collection

The data collection was carried out between January and August 2023 using two sources: a literature review, and interviews with key stakeholders involved in ABR management in Senegal.

Firstly, a literature review was conducted to compile a description of the politico-institutional framework for ABR management at regional and national levels, through a characterization of the stakeholders and of the legal instruments they have developed and/or are applying (policy, regulation, guidelines) to manage ABR. Documents were retrieved by searching the website of the Senegalese government's Official Journal (*Journal Officiel*, https://www.vie-publique.sn/journal-officiel-senegal), and the FAO legal database FAOLex

(https://www.fao.org/faolex). In addition, we consulted the official websites of organizations involved in ABR governance at continental and regional levels (the Africa Centre for Disease Control and Prevention of the African Union, the Regional Animal Health Centre, and the West African Health Organization of the Economic Community of West African States). Additional documents were also suggested by ~~participants to the interview~~. In total, 64 documents were selected for analysis.

In addition, we conducted semi-structured interviews with key stakeholders involved in ABR management to enrich our understanding of the existing politico-institutional framework and to collect their perspectives about One Health governance of ABR in Senegal. The recruitment of participants was conducted from 26/01/2023 to 30/09/2023 in two steps, as described by Laumann *et al.* (1983) [26]. First, we established an initial list of participants corresponding to the members of the thematic working group for AMR of the national One Health platform (67 people, each representing one institution). Then, we asked three experts in the field of ABR management in Senegal to revise the list. We ended up with 28 target participant institutions, which was extended to 29 during data collection as another key institution was mentioned during an interview. Official invitations were sent to the heads of each of the 29 institutions, who were requested to designate their most knowledgeable representative(s) with regard to the theme at hand. Semi-structured interviews were carried out using an interview guide consisting of six parts, all related to ABR management in Senegal: (1) the respondent's characteristics (professional background, role and missions in his/her institution); (2) the role of his/her institution in policy development and implementation; (3) the description of the national policy landscape; (4) the public policy development process (participation and modalities); (5) the implementation and enforcement of public policy; (6) the surveillance of antibiotic use and ABR; and (7) the coordination mechanisms for multi-sectoral governance (existing and potential future mechanisms). In total, we interviewed 37 people across 26 interviews, 92

percent of which were recorded. Interviews were conducted face-to-face in 90 percent of cases, and lasted between 60 and 90 minutes. All interviews were manually transcribed, with repeated listening and proofreading to ensure quality. Unrecorded interviews were faithfully reconstructed on the basis of written notes. Participants belonged to: governmental authorities (13 people from seven institutions); scientific and technical institutions (14 people from 11 institutions); regional and international organizations (eight people from six institutions, three at regional level and three at international level); and the private sector (two informants from two institutions). The participants worked in the animal health sector (11), the agriculture and food sector (11), the human health sector (eight), the ~~environment~~ sector (three). Four participants operated in a multi-sectoral organization.

This study was approved and authorized by the National Ethics Committee for Health Research (*Comité National d'Ethique pour la Recherche en Santé*, or CNERS) in January 2023. Before the start of each interview, informants were informed of this approval in addition to detailed information about the study objectives. In addition, written informed consent was requested and anonymity in data processing was guaranteed to each participant.

Data analysis

Data analysis was carried out in two phases.

Firstly, we conducted a qualitative descriptive analysis of the contents of the documents retrieved during the literature review, and of other documents mentioned during the interviews, in order to characterize the politico-institutional framework, i.e.: (i) the institutions involved in ABR governance (sector, mission, institutional level); (ii) the legal instruments related to ABR (nature, implementation, intervention domain); and (iii) the surveillance programmes for antibiotic use, resistance and residues (regulatory status, objective, strategy). The attributes

used to describe institutions and instruments were primarily retrieved from the literature [27] and refined during the analysis of the ~~document contents~~ and ~~interview contents~~.

Secondly, a thematic analysis was carried out on the transcribed discourses of the informants, using the Nvivo software (version14) in order to (i) deepen the characterization of the politico-institutional framework obtained with the document analysis, and (ii) identify the One Health capacities for ABR governance in Senegal and orientations for its improvement. The thematic analysis to investigate the One Health governance capacities used an inductive approach, following the process described by Braun and Clarke (2006): (i) familiarization with the data; (ii) generation of initial codes; (iii) generation of initial themes; (iv) review of the themes; (v) definition and naming of the themes; and (vi) writing up [28]. We also mobilized the analytical approach of Ritchie *et al*. (2002) to identify barriers and determinants to effective One Health governance for ABR management and to formulate theme names [29]. The themes were refined following various discussions between members of the research team.

Ethics approval and consent to participate

This study was approved and authorized by the Senegalese National Ethics Committee for Health Research (*Comité National d'Ethique pour la Recherche en Santé*, or CNERS) in January 2023 (N°019MSAM/CNERS/SP). Before the start of each interview, participants were informed of this approval in addition to detailed information about the study objectives. In addition, informed consent was requested and anonymity in data processing was guaranteed in the case of each participant.

Results

Following the analysis of the 64 documents retrieved and of the discourse of the 26 institutions interviewed, we analysed the historical development of the politico-institutional framework of ABR management in Senegal, the diversity of stakeholders involved in the policy-making process, and the necessary changes to move towards a more effective One Health governance of ABR in Senegal.

The politico-institutional framework for antibiotic resistance management in Senegal

Since the early 2000s, Senegal has been developing a politico-institutional framework to support the management of ABR, both at sectoral and multi-sectoral levels, in line with international standards (Figure 1).

Fig. 1 Milestones of the development of the politico-institutional landscape for the management of antibiotic resistance in Senegal.

AMR = antimicrobial resistance

The National Programme to Combat Nosocomial Infections (*Programme National de Lutte contre les Infections Nosocomiales*, or PRONALIN) was a 10-year strategic plan (2005-2015) launched by the Ministry of Public Health in 2004, focusing on infections caused by resistant bacteria. It led to the establishment of committees to combat healthcare-associated infections in all hospitals and health centres. In 2012, under the PRONALIN framework, a department in charge of laboratories was established by the Ministry of public health to coordinate surveillance activities conducted by laboratories operating in the human, animal, and trade sectors. In 2017, this department led the development of the first national action plan under a One Health approach for a five-year period (2018-2022), in line with the Global Action Plan to

combat AMR issued in 2015 by the WHO. The objective was to provide an effective response, through a One Health approach, to the threat of AMR, including ABR. This policy, developed by technical departments of ministries in charge of human and animal health, never obtained an official approbation by the ministries, which compromised its effective implementation. However, legal instruments were issued to support the implementation of some of the measures included in the national action plan.

With the support of international partners (e.g., WHO and FAO), ~~Senegal established in 2017~~ a national multi-sectoral platform, named the National High Council for Global Health Security (*Haut Conseil national de la sécurité sanitaire mondiale*), under the Prime Minister. This national One Health platform is in charge of defining the strategic orientations of the One Health global health security programme [30] in compliance with the International Health Regulations (IHR) [31]. It includes a permanent secretary and 14 thematic working groups, aligned with the main domains addressed by the IHR, one of which is dedicated to AMR. These groups bring together representatives of the different ministries, academia, technical and financial partners (TFPs), decentralized authorities, professional organizations and civil society. The national One Health platform is currently in a process of decentralization, with the establishment of similar institutional arrangements at the regional level (the One Health Regional Committees), under the authority of the governors. So far, these regional platforms are barely operational, except in the region of Saint Louis where a regional One Health action plan was developed in 2024. In 2020, the AMR thematic working group of the national One Health Platform drafted an AMR stewardship plan, under the impetus of the Medicines, Technologies, and Pharmaceutical Services project financed by the United States Agency for International Development (USAID), which at the time of writing has been barely operationalized. In October 2022, this same group took the lead to formulate the second national action plan to combat AMR, that was finalized in March 2024. Following the orientations of

the IHR, the national action plan sets key priorities for combating AMR. The development of this second plan also allowed the country to comply with one criterion assessed by the Joint External Evaluation (JEE), the tool developed by the WHO to evaluate the level of compliance of the countries regarding the IHR provisions.

The Senegalese politico-institutional framework is also embedded in the continental and regional one. The African Union has issued several instruments to harmonize AMR management across its member countries, such as an action plan [32], a communication plan [33], and an antimicrobial surveillance framework [34]. The Economic Community of West African States has established a Regional One Health platform, which is currently barely functional, that brings together the Regional Animal Health Centre and the West African Health Organization, and plays a role in the harmonization of national policies and regulations at the regional scale. The Regional Drug Committee of the West African Economic and Monetary Union has issued regulations related to marketing authorization for medicinal products, including antibiotics, for animal health (2006) and human health (2020), allowing the monitoring of products' circulation and ensuring their quality in the region.

A representation of the Senegalese institutional framework in the international and regional context is presented in Figure 2.

Fig. 2 The Senegalese multi-sectoral institutional framework involved in the management of antibiotic resistance and embedded in the global landscape.

In addition to the national action plans, operational legal instruments (41) have also been developed over the years in the different sectors. They include 30 binding legal instruments such as policies (three), laws (14), decrees (five), orders (five), circulars (two), and directive (one), as well as standards and guidelines (11), that target the prevention of ABR (17), or the

surveillance of antibiotic use and resistance (10), the use of antibiotics (three). They are mainly found in the human (14) and the livestock ~~sector~~ (11) but some have also been issued in the ~~environment~~ sector (seven), at multi sectoral level (seven), and the agriculture and food sector (two). Their implementation and enforcement involved a wide range of stakeholders, such as governmental authorities (nine), national technical and research institutes (seven), professional organizations (four), or the private sector (three). Regarding the prevention of ABR, emphasis is put on the implementation of biosecurity measures and good hygiene practices. The instruments for antibiotic use focus on regulating their access, providing guidelines for their judicious use, or controlling their promotion. In addition, the country counts eight public surveillance programmes for ABR (two), ABU (three), antibiotic residues (two), and antibiotic quality (one). ~~They~~ are operating with very little interconnectedness but efforts are ongoing to integrate part of the data they are generating.

Institutions and their main legal instruments are described in Supplementary Material 1, and the surveillance programmes in Supplementary Material 2.

The One Health governance for antibiotic resistance in Senegal

The analysis of the interview contents with regard to the existing politico-institutional framework allowed us to describe the national capacity in managing the ABR issue from a One Health perspective and to identify opportunities to move towards more effective One Health governance of ABR. We identified five determinants that influence the One Health governance capacities for ABR management in Senegal.

The existence of a multi-sectoral institutional arrangement for the governance of antibiotic resistance

The national One Health platform has contributed significantly to the formulation of multi-sectoral legal instruments related to ABR, including the second national action plan, as illustrated by the following comment from a respondent: "*Since the platform was established, we have transferred to it the tasks of intersectoral development* [of legal instruments]*, as they are strategic, multisectoral and multidisciplinary documents, and therefore they must be truly inclusive and participatory*" (Respondent 17).

However, there are two main obstacles to the platform's effective operation. First, its roles and missions are insufficiently formalized, as indicated by Respondent 5: *"The platform is not very well equipped at the institutional and political level. It still seems to be legally weak. It would be better to strengthen this*". This hampers its ability to mobilize the national stakeholders towards the development of a common vision for the effective management of ABR. Additionally, the platform does not have sufficient domestic budget and human resources to carry out its missions and mainly relies on the financial support of international partners, as described by Respondents 23 and 24: "*If one does not have financial autonomy, it is very difficult to operate. For One Health coordination, you need a team, you need experts in the field.*"; "*As ever, money talks. If you have a very nice car and you don't have the gas, it won't move.*"

If the national One Health capacities for ABR management are to be improved, the platform's legitimacy in relation to sectoral institutions need to be strengthened, and a budget commensurate with its mandate ~~need~~ to be allocated, as suggested by Respondent 1: "*Strengthening the current platform should be enough. It should be enough if they just strengthen the legal framework and ~~to~~ allocate sufficient budget.*"

The influence of the technical and financial partners for the management of antibiotic resistance

The TFPs are very active in the field of ABR management in Senegal. Numerous projects are financed and/or implemented by them in various fields, such as communication about the judicious use of antibiotics (Breakthrough Action project financed by the United States), surveillance of ABR and antibiotic usage (financed by the Fleming Fund), judicious use of antibiotics (programmes financed by the Antimicrobial Resistance Multi-Partner Trust Fund). However, this strong support can have a negative influence on the formulation and implementation of ABR management policies in the long term. Indeed, most programmes initiated and financed by international aid have difficulty surviving when external funding ceases, due to a lack of ownership by the government, which does not take over with domestic resources to maintain them. For instance, a surveillance programme for resistant bacteria in the agri-food sector has been established with FAO support, but national actors do not receive appropriate domestic budgets to keep it running. They are still relying on one-off external fundings to conduct surveillance activities, which need to be carried out in a timely manner, as indicated by a representative: "*Currently, we do not do antibiograms because we don't have any kits. I have made a new request to the FAO. We are waiting and I think they will say yes. They have launched the tender, I am waiting to see"* (Respondent 12). In addition, when formulating policies, national actors tend to prioritize interventions that will attract the attention of donors or that comply with their priorities and agendas: "*When policy is formulated, if activities are proposed that are not considered by or are not in the area of interest of the partners, then [those activities] will not come to much. The key is to be supported by technical and financial partners*" (Respondent 3).

The legal framework shaping the governance of antibiotic resistance

Relevant, comprehensive and enforced legal instruments in all sectors are necessary to establish effective One Health governance of ABR. Senegal understood very early on the importance of having a legal framework to maintain the effectiveness of antibiotics, one that regulates their importation, distribution and use. However, the country's normative framework faces major problems. First, some provisions of the prevailing legal instruments are outdated regarding the rapidly changing context of ABR emergence and spread, as related by Respondent 3: *"There is a weakness in the regulation compared to these provisions* [on the detention and use of antibiotics]. *Because this* [ABR] *is an emerging phenomenon that has not been sufficiently taken into account*." This shortcoming is particularly alarming for the regulation on the use of antibiotics, as antibiotics can be accessed freely even by people who are not entitled to them: *"The regulations are not precise and there is no specification of standards so people deliver drugs without prescription and practice "Kalashnikov treatments"* [meaning that they use critically important antibiotics to treat mild infections]*)"* (Respondent 8). Furthermore, although the institutions responsible for implementing legal instruments are well-defined and have the necessary powers, they ~~a~~ lack ~~of~~ human and financial resources to carry out their supervisory and control tasks, as described by Respondent 21*: "For veterinary drug inspection, there are only three specialized inspectors at the national scale. It is very insufficient and, even worse, the logistical and financial means are insufficient.*" Moreover, the application and enforcement of the law is hampered by passive corruption that is favoured by friendly or family relations: *"Unfortunately, we lack seriousness in the application of these texts. We have a term for that, called the* massla: *if I'm friends with someone and he is doing wrong things, I will*

consider it is ok, and this culture is holding us back [in the application of laws]*"* (Respondent 22).

Moving towards more effective ABR management requires the updating and enforcement of the prevailing legal frameworks in different sectors, as suggested by Respondent 9: *"We need to update certain regulatory provisions among the ones existing and ensure that they are applied. This will enable us to combat ABR effectively.*"

The capacity of the healthcare system

Senegal's healthcare system has improved significantly, particularly following the various health crises that have affected the country (Ebola, COVID-19), with a particular focus on equity and inclusiveness. More specifically, committees in charge of combatting nosocomial infections have been established in hospitals to improve infection prevention and hygiene in healthcare environments, with a focus on ABR. However, the healthcare system is not appropriately equipped at national scale to contribute effectively to the fight against ABR. In some remote areas, no bacteriology laboratories are available in either the human or the animal sector. Where laboratory facilities exist, they face shortages of reagents for lab testing. As a result, few diagnostic results are available to guide the judicious prescription of antibiotics; practitioners may use antibiotics when not needed or the wrong class of antibiotics for a particular bacterial strain (for instance critical antibiotics for benign infections). This is reported by Respondent 15: "*Not all hospital facilities are well equipped with laboratories that can carry out treatments based on antibiograms. Consequently, treatment is probabilistic."* This lack of diagnostic capacities also impacts the quality of the surveillance data, as underlined by Respondent 14: "*It's clear that there are bacteria in remote areas, and there are resistances there. But if we don't have a bacteriology laboratory capable of carrying out sensitivity tests, this information escapes us.*"

To be able to properly manage ABR, Senegal must strengthen its capacity in terms of laboratory diagnostics to improve the judicious use of antibiotics and to generate ABR surveillance data to appropriately inform the policy-making process. Among other things, this requires building capacity to conduct laboratory testing at national scale, as suggested by Respondent 8: *“A staff training programme would enable laboratories in the country to have the capacity to carry out sensitivity tests that would at least make it possible to know what type of antibiotic to use or whether or not a bacteria is resistant.*” This would also require the official appointment of an institution capable of collating all the data produced in the country. For the human sector, the national public health laboratory could play this role, as described in the case of Nigeria by Respondent 23: “*In Nigeria, there is only one structure that manages everything. The CDC* [Centre for Disease Control and Prevention] *manages all surveillance, the laboratory, the response and the fight against ABR, and all the information converges there.”*

The power balance among stakeholders

In recent years, national stakeholders have developed substantial experience in both formal and informal collaboration for managing health issues with a One Health perspective, particularly in the context of COVID-19 and other epidemics. However, this collaboration runs into power issues between stakeholders. First, some professions operating in the human health sector, and to a lesser extent those working in the animal health sector, consider that they have a monopoly when it comes to public health, as observed by Respondent 22: “*During these workshops* [for the formulation of the second national action plan*], each corporation pulls on its side; that is to say, if we talk about One Health, for example, the* [medical] *doctors always want to be the profession who leads. We forget about the environmentalist, we forget about the veterinarian, we forget about the pharmacist.*” They tend to overlook the points of view and perspectives of other professions, which are usually less directly involved in public health, and this hampers

the design of collective solutions for an effective management of ABR. Respondent 12 recognized that, during the drafting of the second national action plan, "*a certain censorship is to be seen, which can effectively block the whole group's progress.*" Power issues were also described in the field of ABR surveillance, when it comes to sharing data across professions and sectors in an effort to provide the most comprehensive information to support the decision-making process. Data is a valuable asset for stakeholders, either to valorise them in scientific papers - which are important for recognition and promotion within the scientific community - or for showcasing their work to attract funding from international aid. Consequently, "*there is this perception that each sector tends to keep its information* [surveillance data] *for itself, and that's a brake to the operationalization of the AMR surveillance system"* (Respondent 8). Interview contents suggested that there was a need to better define the roles and responsibilities of the various stakeholders in the national One Health surveillance system, as mentioned by Respondent 24: "*It will be sufficient to have a regulatory framework that lays out what data is to be collected, how it will be collected, and which institutions are to be involved in the management of data.*"

Discussion

By combining a review of legal documents and interviews with stakeholders, this study provides an overview of the political-institutional framework for the governance of ABR in Senegal, and of its capacity to manage ABR using a One Health approach. Since 2017, Senegal has been committed to an integrated, cross-sectoral approach to ABR. Although this is only partially functional, Senegal already has multi-sectoral institutional mechanisms at central and local levels to guide and monitor ABR-related policy implementation. An updated national

policy to combat ABR exists, but its operational implementation remains partial. The regulatory framework is comprehensive, covering the areas of prevention, use and monitoring, but is not necessarily always appropriately localized or enforced. As ABR is a major international concern, the country receives considerable technical and financial support from external partners, particularly international organizations such as the FAO and WHO and development agencies such as USAID, to develop and implement ABR-related policies. This external influence can compromise its abilities to develop policy solutions aligned on national priorities and a shared vision to address the ABR issue.

The evaluation of Senegal's capacities to manage ABR from a One Health perspective is based on the perception and posture of the different categories of actors involved in ABR governance in the country. However, some major actors in the human health sector declined to participate in the study. Furthermore, ~~and~~ we cannot certify that the discourse of those who did participate was a typical representation of the views of their institution.

The politico-institutional landscape to combat antibiotic resistance in sub-Saharan African countries

In recent decades, African countries have developed inter-sectoral institutional mechanisms, commonly known as national One Health platforms, to better manage health issues at the human-animal-environment interface, such as zoonoses and ABR. The development of these mechanisms has been accelerated by the succession of various health crises (e.g. Ebola) and largely encouraged by the TFPs, in particular the Tripartite[1] with the financial support of USAID and the World Bank through the REDISSE (Regional Disease Surveillance Systems Enhancement) project [36, 37, 38]. ~~All these~~ platforms have more or less the same organizational structure, with a permanent secretariat and thematic groups modelled on the

IHR's technical areas, including one dedicated to AMR. However, they differ in their governance arrangements. In some countries, such as Senegal, they are headquartered at the highest level of government, in the Prime Minister's Office. In others, they are hosted within a ministry (usually the one responsible for public health, as in Burkina Faso or Kenya), with a more-or-less rotating secretariat among sectors, as is the case in Uganda [40 , 41 , 42 , 43]. Some countries have begun to decentralize these mechanisms to the local level (Kenya and Senegal). These platforms have undeniably improved the governance of complex health hazards, by providing a forum for discussion and exchange for all stakeholders, including governmental authorities, professionals, technical and scientific institutes and the civil society [44 , 45]. However, they face a number of difficulties, which vary in severity from country to country, mainly: (i) a lack of domestic resources, which makes them highly dependent on external resources and compromises their autonomy and sustainability [42]; (ii) a lack of legal framework clearly defining their roles and missions and of the political support, which brings confusion regarding the platform’s leadership for orienting and implementing One Health interventions; (iii) a lack of a strategic vision taking into account concerns and priorities of the whole array of stakeholders, including actors that are not traditionally considered to be at the centre of global health issues [43]; and (iv) a lack of specific capacities in managing multi-stakeholder platforms, in policy development and evaluation, and in facilitating science-society-policy dialogue [44]. In many countries, the tasks assigned to the platforms have come to duplicate the national epidemic management committees already in place, resulting in the dilution of already limited resources. During the COVID-19 pandemic, specific committees were established to coordinate health actions instead of mobilizing the existing platform to take charge of this task. This calls into question the legitimacy of these platforms during the management of health crises [45].

Additionally, following the adoption of the Global Action Plan for AMR in 2015, a large number of African countries, including Senegal, with the strong support of TFPs, have developed their own action plan for AMR. In total, 90 percent of all countries, and 61 percent of LMICs, have declared a National Action Plan for AMR in 2023 [46]. However, implementing actions described in these policies remain a real challenge for these countries, mainly because they are not adapted to their resources, priorities and needs [48 , 49].
Consequently, as the establishment of national One Health platforms was more a reaction to a financing opportunity than to a shared multisectoral problem, these institutions can be seen as aid recipients rather than real strategic governance instruments with institutional power and appropriate domestic fundings.

The institutionalization of One Health governance for antibiotic resistance management

The results of this study raise the question of how to improve One Health governance of ABR, in the specific context of LMICs such as Senegal, where the pressure of ABR is particularly high and the resources to curb it are limited [47]. A solution often put forward by the scientific community and development actors is to strengthen the institutionalization of One Health governance, at both the global and local levels [51 , 52 , 53 , 54]. By institutionalization, authors refer to the establishment of institutional arrangements backed up by a political support and a legal framework specifying the role and missions of all stakeholders for the production and translation of knowledge, and the development, implementation and evaluation of policies, to achieve a collectively defined global vision. Indeed, strengthening institutionalization suggests more effective governance by reinforcing certain key aspects, such as participation, collaboration and coordination, accountability and transparency, sustainability, effective

management and equity [52]. However, the institutionalization of One Health governance is not without risks, at both global and national levels.

At the international level, strengthening the institutionalization of ABR governance would inevitably require the development of global policies to which countries, whatever their socio-economic context, would have to adhere [50]. For ABR, where the different contexts are even more varied than for other health issues [53], the difficulty lies in developing policies that are equitable across countries and populations and that take into account the diversity of socio-economic and political contexts [52]. The historical prioritization of the needs of high-income countries over those of LMICs in global policies risks leading to the development of ABR-related policies that focus on the benefits for a restricted fraction of the world, such as policies focusing on the conservation of critical antibiotics at the expense of improving access to antibiotics and associated mortality [59 , 60 , 61], and that can be implemented only in contexts that show comprehensive legal frameworks, adequate enforcement capacities, and low corruption levels [57]. Consequently, to comply with international standards, or influenced by TFP-organized workshops, countries with limited resources can find themselves compelled to adopt ABR-related policies with unattainable objectives that are ill-suited to their implementation capacity or to their needs and priorities [58]. Additionally, as TFPs align their support with global policies, there is always a risk that a given government will be deprived of funding if it does not align itself with the priorities of international funders [59].

Even when not binding, these global policies and strategies have a strong influence on national policy frameworks. Indeed, they are often accompanied by evaluation tools, such as the JEE that assesses the country compliance with the IHR and the WOAH’s norms, or the FAO Progressive Management Pathway for AMR (FAO-PMP-AMR) that ~~monitors the~~ implementation of the FAO Action Plan on AMR 2021-2025 ~~at country level~~. The evaluations carried out in the countries using these tools to check their level of compliance with the related

policies lead to evidence and recommendations that are then used by TFPs to plan their future support to the country, or by the countries themselves to propose interventions that are more likely to be attractive for donors. This leaves little room for manoeuvre for countries to develop policy solutions and interventions more tailored to the local context, for instance by engaging with populations affected by ABR or with authorities operating at the most local level over policy design [56 , 60]. Additionally, these evaluation tools also place countries in a race for ratings, sometimes to the detriment of other national priorities. Indeed, good scores makes them more attractive than countries with lower scores for the implementation of programmes to supporting the fight against ABR [57 , 58].

At the national level, institutionalization of One Health governance for the management of ABR is not a guarantee of efficiency. While binding legal instruments institutionalizing the roles and responsibilities of actors can improve their participation in certain collective activities, this does not ensure their commitment to working collectively to find and implement political solutions to the ABR issue [61]. Collaboration may feel more of an obligation than an opportunity to share points of view and expertise in the service of collective action. Additionally, people are more reluctant to collaborate when the rules are laid down in a binding document, because they feel they are trapped in interactions from which they cannot withdraw [62]. In Senegal, as in other LMICS, the process of institutionalizing One Health governance through the establishment of new institutional arrangements and policies has not been accompanied by sufficient additional domestic resources. Human and financial resources in technical departments of ministries and in other organizations involved in ABR One Health governance are drastically insufficient to allow engagement of the staff in intersectoral activities without compromising the good implementation of their primary missions. This is exacerbated by the fact that stakeholders are overburdened with a multiplicity of ABR-related activities proposed by different TFPs in a context where international aid is still too often fragmented,

uncoordinated and even in competition [48, 63, 64] and incentivizes the participation of national counterparts [55]. Countries rely mainly on external funding to develop, implement and evaluate ABR-related policies, and this makes them vulnerable in terms of political sovereignty as they have to shape their politico-institutional framework in light of the funding available. Additionally, as this funding is not sustainable over time because it is backed by short-term programmes, the politico-institutional framework may be disrupted when these programmes come to an end, because domestic funding is unable to take over [41, 42, 55]. Most of the One Health initiatives across Africa are funded by international aid, with some partial co-funding from national governments [64]. In Senegal, the One Health platform's annual work programmes and budget for 2024 includes 189 activities, of which only a small proportion is either funded (2%) or co-funded by the state (17%). In this context of scarce resources, the institutionalization of One Health governance can generate competition between actors working in the technical directorates of the Ministries and the national One Health platform. Additionally, part of the technical and financial aid that used to go directly to sectoral ministries is now channelled through the national One Health platform, creating frustration among some actors who are refocusing on their sectoral activities.

Consequently, this shift towards the reinforcement of institutionalization of One Health governance ~~strengthen~~ the powers of global players, with the creation of competitive ~~space~~ at the international and national levels [65].

Conclusions

In a health context calling for One Health governance in order to combat the problem of ABR, Senegal has established a politico-institutional framework aligned with international standards. However, strengthening the legal framework, improving healthcare infrastructure and

enhancing the capacity of the interesctoral institutional arrangements is critical to effectively manage the ABR issue from a One Health perspective.

One Health governance is foundational in developing, improving, and sustaining ABR-related policies given the complex nature of the emergence and spread of ABR and the structural political-economic factors that drive its management. Institutionalization is necessary to ensure collaboration and coordination, accountability and transparency, equity, participation, effective management, and sustainability, which are key attributes of effective One Health governance. However, institutionalization is not without risk. At a global level, strengthening global policies and the management tools can have an impact on the relevance and sustainability of the politico-institutional frameworks within countries. At a national level, it can lead to competition and a dispersal of resources between actors, which is detrimental to the collective action that underpins One Health governance. A strengthening of the institutionalization of One Health governance for ABR must be accompanied by a reform of the process of developing and implementing global and national policies for a better representation of interests and priorities. Failing to do so may consolidate power in the hands of few stakeholders at international and national level and to inequity between sectors and populations with regard to the positive and negative impacts of these policies.

Endnotes

[1] The Tripartite consisted of the first three members of the current Quadripartite mentioned on page 3, namely the WHO, the Food and Agriculture Organization (FAO), and the World Organization for Animal Health (WOAH), from 2010 to 2022, after which the United Nations Environment Programme (UNEP) joined as a fourth member.

Acknowledgements

The authors would like to thank all the participants, and Kadiatou Barry who helped with the transcriptions of the interviews.

References

1. World Health Organization. Antimicrobial resistance. [cited 27 May 2024]. Available: https://www.who.int/news-room/fact-sheets/detail/antimicrobial-resistance

2. O'Neil J. Tackling drug-resistant infections globally: final report and recommendations. 2016. Accessed on: May 27 2024. Available at: https://apo.org.au/sites/default/files/resource-files/2016-05/apo-nid63983.pdf.

3. Murray CJL, Ikuta KS, Sharara F, Swetschinski L, Aguilar GR, Gray A, et al. Global burden of bacterial antimicrobial resistance in 2019: a systematic analysis. The Lancet. 2022;399: 629–655. doi:10.1016/S0140-6736(21)02724-0

4. Krockow EM, Tarrant C. The international dimensions of antimicrobial resistance: Contextual factors shape distinct ethical challenges in South Africa, Sri Lanka and the United Kingdom. Bioethics. 2019;33: 756–765. doi:10.1111/bioe.12604

5. Elton L, Thomason MJ, Tembo J, Velavan TP, Pallerla SR, Arruda LB, et al. Antimicrobial resistance preparedness in sub-Saharan African countries. Antimicrob Resist Infect Control. 2020;9: 145. doi:10.1186/s13756-020-00800-y

6. Endale H, Mathewos M, Abdeta D. Potential Causes of Spread of Antimicrobial Resistance and Preventive Measures in One Health Perspective-A Review. Infect Drug Resist. 2023;16: 7515–7545. doi:10.2147/IDR.S428837

7. Ahmed SK, Hussein S, Qurbani K, Ibrahim RH, Fareeq A, Mahmood KA, et al. Antimicrobial resistance: Impacts, challenges, and future prospects. J Med Surg Public Health. 2024;2: 100081. doi:10.1016/j.glmedi.2024.100081

8. Queenan K, Häsler B, Rushton J. A One Health approach to antimicrobial resistance surveillance: is there a business case for it? Int J Antimicrob Agents. 2016;48: 422–427. doi:10.1016/j.ijantimicag.2016.06.014

9. McEwen SA, Collignon PJ. Antimicrobial Resistance: a One Health Perspective. Antimicrobial Resistance in Bacteria from Livestock and Companion Animals. John Wiley & Sons, Ltd; 2018. pp. 521–547. doi:10.1128/9781555819804.ch25

10. Holmes AH, Moore LSP, Sundsfjord A, Steinbakk M, Regmi S, Karkey A, et al. Understanding the mechanisms and drivers of antimicrobial resistance. The Lancet. 2016;387: 176–187. doi:10.1016/S0140-6736(15)00473-0

11. Samreen, Ahmad I, Malak HA, Abulreesh HH. Environmental antimicrobial resistance and its drivers: a potential threat to public health. J Glob Antimicrob Resist. 2021;27: 101–111. doi:10.1016/j.jgar.2021.08.001

12. Facteurs des problèmes émergents en matière de santé animale et végétale - Richardson - 2016 - EFSA Journal - Wiley Online Library. [cited 27 May 2024]. Available: https://efsa.onlinelibrary.wiley.com/doi/full/10.2903/j.efsa.2016.s0512

13. Frost I, Van Boeckel TP, Pires J, Craig J, Laxminarayan R. Global geographic trends in antimicrobial resistance: the role of international travel. J Travel Med. 2019;26: taz036. doi:10.1093/jtm/taz036

14. Kotwani A, Joshi J, Kaloni D. Pharmaceutical effluent: a critical link in the interconnected ecosystem promoting antimicrobial resistance. Environ Sci Pollut Res. 2021;28: 32111–32124. doi:10.1007/s11356-021-14178-w

15. World Health Organization (WHO). Global action plan on antimicrobial resistance. 2016. Accessed on: October 2 2023. Available at: efaidnbmnnnibpcajpcglclefindmkaj/https://apps.who.int/gb/ebwha/pdf_files/WHA69/A69_24-en.pdf.

16. So AD, Shah TA, Roach S, Ling Chee Y, Nachman KE. An Integrated Systems Approach is Needed to Ensure the Sustainability of Antibiotic Effectiveness for Both Humans and Animals. J Law Med Ethics J Am Soc Law Med Ethics. 2015;43 Suppl 3: 38–45. doi:10.1111/jlme.12273

17. Zinsstag J, Meisser A, Schelling E, Bonfoh B, Tanner M. From “two medicines” to “One Health” and beyond. Onderstepoort J Vet Res. 2012;79: 492. doi:10.4102/ojvr.v79i2.492

18. World Health Organization: Tripartite and UNEP support OHHLEP’s definition of “One Health.” [cited 27 May 2024]. Available: https://www.who.int/news/item/01-12-2021-tripartite-and-unep-support-ohhlep-s-definition-of-one-health

19. World Health Organization - 2015 - Global action plan on antimicrobial resistance.pdf. Available: https://iris.who.int/bitstream/handle/10665/193736/9789241509763_eng.pdf

20. Harant A. Assessing transparency and accountability of national action plans on antimicrobial resistance in 15 African countries. Antimicrob Resist Infect Control. 2022;11: 15. doi:10.1186/s13756-021-01040-4

21. Food and Agriculture Organization (FAO). Action Plan on Antimicrobial Resistance2016-2020. 2016. Accessed on: October 2 2023. Available at: http://www.fao.org/3/a-i5996e.pdf

22. World Organization of Animal Health (WOAH). The OIE Strategy on Antimicrobial Resistance and the Prudent Use of Antimicrobials. 2016. Accessed on: October 2nd 2023. Available at: http://www.oie.int/antimicrobial-resistance

23. Birgand G, Castro-Sánchez E, Hansen S, Gastmeier P, Lucet J-C, Ferlie E, et al. Comparison of governance approaches for the control of antimicrobial resistance: Analysis of three European countries. Antimicrob Resist Infect Control. 2018;7: 28. doi:10.1186/s13756-018-0321-5

24. Pierre J, Carelli D, Peters BG. The four worlds of politics and administration in the EU: how institutional arrangements shape the struggle against antimicrobial resistance. J Eur Public Policy. 2023;0: 1–28. doi:10.1080/13501763.2023.2255223

25. Seck M, Seck I, Sow AI, Ndoye B, et al. Governance against antimicrobial resistance in Africa: coping with limited resources, the example of Senegal. Accessed on: January 9 2023. Available at: http://resistancecontrol.info/wp- content/uploads/2018/05/10-15.pdf

26. Laumann E, Marsden P, Prensky D. The Boundary Specification Problem in Network Analysis. Appl Netw Anal Methodol Introd. 1983;61.

27. Garza M, Mohan CV, Brunton L, Wieland B, Häsler B. Typology of interventions for antimicrobial use and antimicrobial resistance in aquaculture systems in low- and middle-income countries. Int J Antimicrob Agents. 2022;59: 106495. doi:10.1016/j.ijantimicag.2021.106495

28. Braun V, Clarke V. Using thematic analysis in psychology. Qual Res Psychol. 2006;3: 77–101. doi:10.1191/1478088706qp063oa

29. Long Q, Jiang H. Qualitative research in health: value and visibility. Lancet Reg Health – West Pac. 2023;34. doi:10.1016/j.lanwpc.2023.100790

30. United States Agency for international Development. Global Health Security Agenda (GHSA). Framework 2028. 2024. Accessed on: October 12 2024. Available at: https://globalhealthsecurityagenda.org/ghsaframework

31. World Organization Health (WHO). International Health Regulations (2005). [cited 20 Oct 2024]. Available: https://www.who.int/publications/i/item/9789241580410

32. African Union. African Union Framework for Antimicrobial Resistance Control 2020–2025. 2021. Accessed on: March 10 2024. Available at: https://africacdc.org/download/african-union-framework-for-antimicrobial-resistance-control-2020-2025

33. African Union. Africa Regional Strategy on Antimicrobial Resistance Communications and Advocacy. FAO; UNEP; WHO; World Organisation for Animal Health (WOAH) (founded as OIE); 2022. doi:10.4060/cc2576fr

34. Africa Centers for Disease Control and prevention (CDC). Africa CDC Framework for Antimicrobial Resistance. 2018. Accessed on: March 10 2024. Available at: https://africacdc.org/download/africa-cdc-framework-for-antimicrobial-resistance/

35. Hussein HA. Brief review on ebola virus disease and one health approach. Heliyon. 2023;9: e19036. doi:10.1016/j.heliyon.2023.e19036

36. Munyua PM, Njenga MK, Osoro EM, Onyango CO, Bitek AO, Mwatondo A, et al. Successes and challenges of the One Health approach in Kenya over the last decade. BMC Public Health. 2019;19: 465. doi:10.1186/s12889-019-6772-7

37. Okello AL, Bardosh K, Smith J, Welburn SC. One Health: Past Successes and Future Challenges in Three African Contexts. PLoS Negl Trop Dis. 2014;8: e2884. doi:10.1371/journal.pntd.0002884

38. Mwatondo A, Munyua P, Gura Z, Muturi M, Osoro E, Obonyo M, et al. Catalysts for implementation of One Health in Kenya. Pan Afr Med J. 2017;ARTVOL. doi:10.11604/pamj.supp.2017.28.1.13275

39. Agbo S, Gbaguidi L, Biliyar C, Sylla S, Fahnbulleh M, Dogba J, et al. Establishing National Multisectoral Coordination and collaboration mechanisms to prevent, detect, and respond to public health threats in Guinea, Liberia, and Sierra Leone 2016–2018. One Health Outlook. 2019;1: 4. doi:10.1186/s42522-019-0004-z

40. Buregyeya E, Atusingwize E, Nsamba P, Musoke D, Naigaga I, Kabasa JD, et al. Operationalizing the One Health Approach in Uganda: Challenges and Opportunities. J Epidemiol Glob Health. 2020;10: 250–257. doi:10.2991/jegh.k.200825.001

41. Fasina FO, Bett B, Dione M, Mutua F, Roesel K, Thomas L, et al. One Health gains momentum in Africa but room exists for improvement. One Health. 2022;15: 100428. doi:10.1016/j.onehlt.2022.100428

42. Fasina FO, Fasanmi OG, Makonnen YJ, Bebay C, Bett B, Roesel K. The one health landscape in Sub-Saharan African countries. One Health. 2021;13: 100325. doi:10.1016/j.onehlt.2021.100325

43. Khan MS, Rothman-Ostrow P, Spencer J, Hasan N, Sabirovic M, Rahman-Shepherd A, et al. The growth and strategic functioning of One Health networks: a systematic analysis. Lancet Planet Health. 2018;2: e264–e273. doi:10.1016/S2542-5196(18)30084-6

44. Legido-Quigley H, Khan MS, Durrance-Bagale A, Hanefeld J. Something Borrowed, Something New: A Governance and Social Construction Framework to Investigate Power Relations and Responses of Diverse Stakeholders to Policies Addressing Antimicrobial Resistance. Antibiotics. 2019;8: 3. doi:10.3390/antibiotics8010003

45. Nana SD, Caffin J-H, Duboz R, Antoine-Moussiaux N, Binot A, Diagbouga PS, et al. Towards an integrated surveillance of zoonotic diseases in Burkina Faso: the case of anthrax. BMC Public Health. 2022;22: 1535. doi:10.1186/s12889-022-13878-3

46. Charani E, Mendelson M, Pallett SJC, Ahmad R, Mpundu M, Mbamalu O, et al. An analysis of existing national action plans for antimicrobial resistance—gaps and opportunities in strategies optimising antibiotic use in human populations. Lancet Glob Health. 2023;11: e466–e474. doi:10.1016/S2214-109X(23)00019-0

47. Ventelou B, Afridi MA, Moatti J-P. Flux internationaux d'aide à la santé, taux de mortalité adulte et PIB:Le « triangle d'or » du développement ? Rev Tiers Monde. 2013;215: 93–110. doi:10.3917/rtm.215.0093

48. Leboeuf A. Making Sense of One Health: Cooperating at the Human-Animal-Ecosystem Health Interface. [cited 9 Sep 2024]. Available: https://www.ifri.org/en/publications/etudes-de-lifri/making-sense-one-health-cooperating-human-animal-ecosystem-health

49. Laxminarayan R, Van Boeckel T, Frost I, Kariuki S, Khan EA, Limmathurotsakul D, et al. The Lancet Infectious Diseases Commission on antimicrobial resistance: 6 years later. Lancet Infect Dis. 2020;20: e51–e60. doi:10.1016/S1473-3099(20)30003-7

50. Hobeika A, Stauffer MHT, Dub T, Bortel W van, Beniston M, Bukachi S, et al. The values and risks of an Intergovernmental Panel for One Health to strengthen pandemic prevention, preparedness, and response. Lancet Glob Health. 2023;11: e1301–e1307. doi:10.1016/S2214-109X(23)00246-2

51. Lee K, Brumme ZL. Operationalizing the One Health approach: the global governance challenges. Health Policy Plan. 2013;28: 778–785. doi:10.1093/heapol/czs127

52. Ruckert A, Harris F, Aenishaenslin C, Aguiar R, Boudreau-LeBlanc A, Carmo LP, et al. One Health governance principles for AMR surveillance: a scoping review and conceptual framework. Res Dir One Health. 2024;2: e4. doi:10.1017/one.2023.13

53. Kirchhelle C, Atkinson P, Broom A, Chuengsatiansup K, Ferreira JP, Fortané N, et al. Setting the standard: multidisciplinary hallmarks for structural, equitable and tracked antibiotic policy. BMJ Glob Health. 2020;5: e003091. doi:10.1136/bmjgh-2020-003091

54. Van Patter LE, Linares-Roake J, Breen AV. What does One Health want? Feminist, posthuman, and anti-colonial possibilities. One Health Outlook. 2023;5: 4. doi:10.1186/s42522-022-00076-9

55. Ridde V, Faye A. Policy response to COVID-19 in Senegal: power, politics, and the choice of policy instruments. Policy Des Pract. 2022;5: 326–345. doi:10.1080/25741292.2022.2068400

56. Siachalinga L, Mufwambi W, Lee I-H. Impact of antimicrobial stewardship interventions to improve antibiotic prescribing for hospital inpatients in Africa: a systematic review and meta-analysis. J Hosp Infect. 2022;129: 124–143. doi:10.1016/j.jhin.2022.07.031

57. Collignon P, Beggs JJ, Walsh TR, Gandra S, Laxminarayan R. Anthropological and socioeconomic factors contributing to global antimicrobial resistance: a univariate and multivariable analysis. Lancet Planet Health. 2018;2: e398–e405. doi:10.1016/S2542-5196(18)30186-4

58. Olivier de Sardan J-P. La revanche des contextes. Des mésaventures de l'ingénierie sociale, en Afrique et au-delà. Karthala Editions;2021. Article in French

59. Brown GW, Rhodes N, Tacheva B, Loewenson R, Shahid M, Poitier F. Challenges in international health financing and implications for the new pandemic fund. Glob Health. 2023;19: 97. doi:10.1186/s12992-023-00999-6

60. Brown GW, Rhodes N, Tacheva B, Loewenson R, Shahid M, Poitier F. Challenges in international health financing and implications for the new pandemic fund. Glob Health. 2023;19: 97. doi:10.1186/s12992-023-00999-6

61. Kelley RE. The power of followership : how to create leaders people want to follow, and followers who lead themselves / Robert E. Kelley. - Vanderbilt University. [cited 11 Sep 2024]. Available: https://catalog.library.vanderbilt.edu/discovery/fulldisplay/alma991019814629703276/01VAN_INST:vanui

62. Olejniczak K, Śliwowski P, Leeuw F. Comparing Behavioral Assumptions of Policy Tools: Framework for Policy Designers. J Comp Policy Anal Res Pract. 2020;22: 498–520. doi:10.1080/13876988.2020.1808465

63. Lee K, Brumme ZL. Operationalizing the One Health approach: the global governance challenges. Health Policy Plan. 2013;28: 778–785. doi:10.1093/heapol/czs127

64. Otu A, Effa E, Meseko C, Cadmus S, Ochu C, Athingo R, et al. Africa needs to prioritize One Health approaches that focus on the environment, animal health and human health. Nat Med. 2021;27: 943–946. doi:10.1038/s41591-021-01375-w

65. Djelic M, Sahlin-Andersson K. Institutional dynamics in a re-ordering world. 1st ed. In: Djelic M-L, Sahlin-Andersson K, editors. Transnational Governance. 1st ed. Cambridge University Press; 2006. pp. 375–397. doi:10.1017/CBO9780511488665.019

Figure 1

Click here to access/download;Figure;Figure1_ milestones_ politico-institutional_landscape .tif

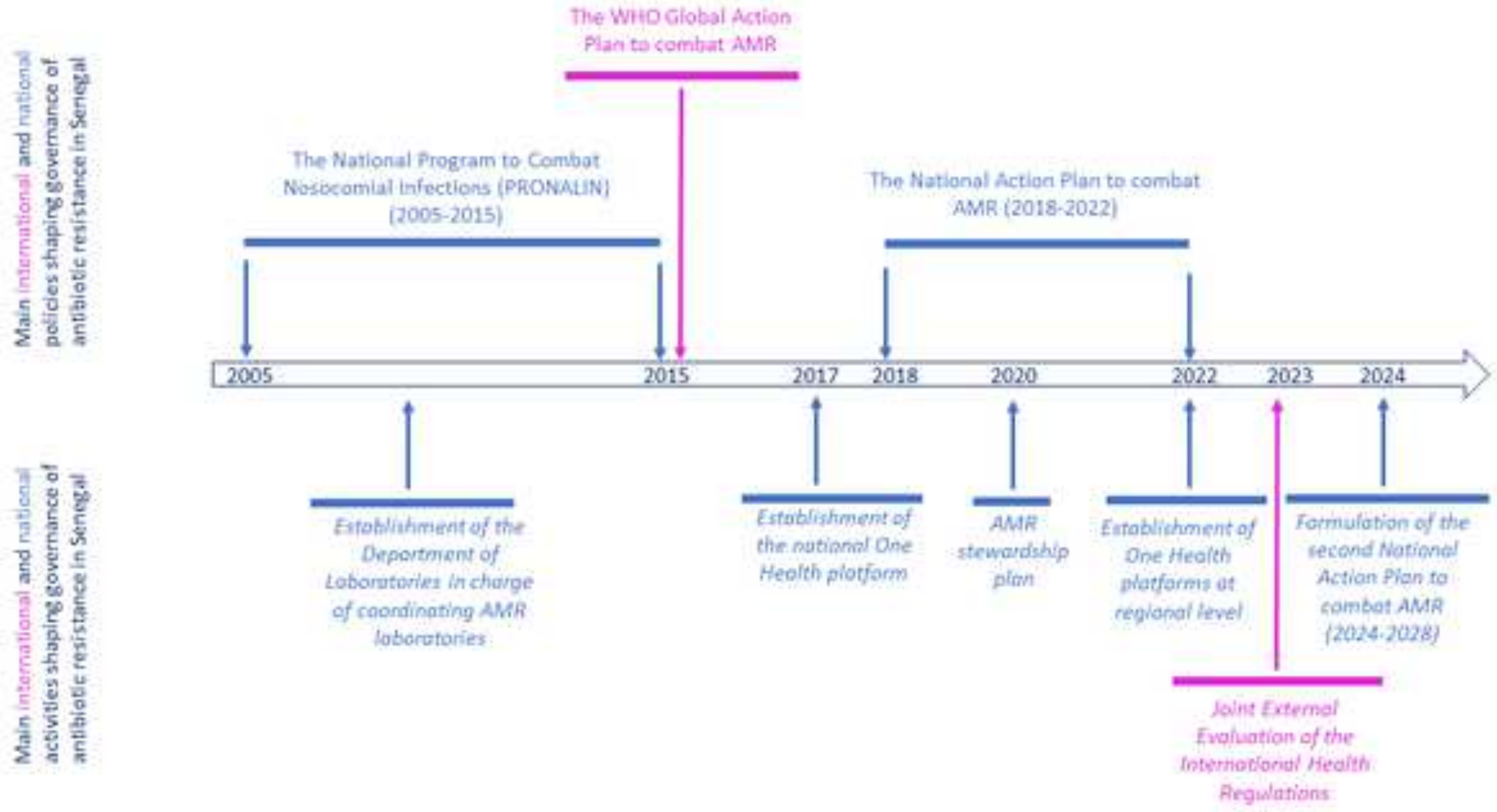

Figure 2

Click here to access/download;Figure;Figure2_institutional_framework_Senegal.tif

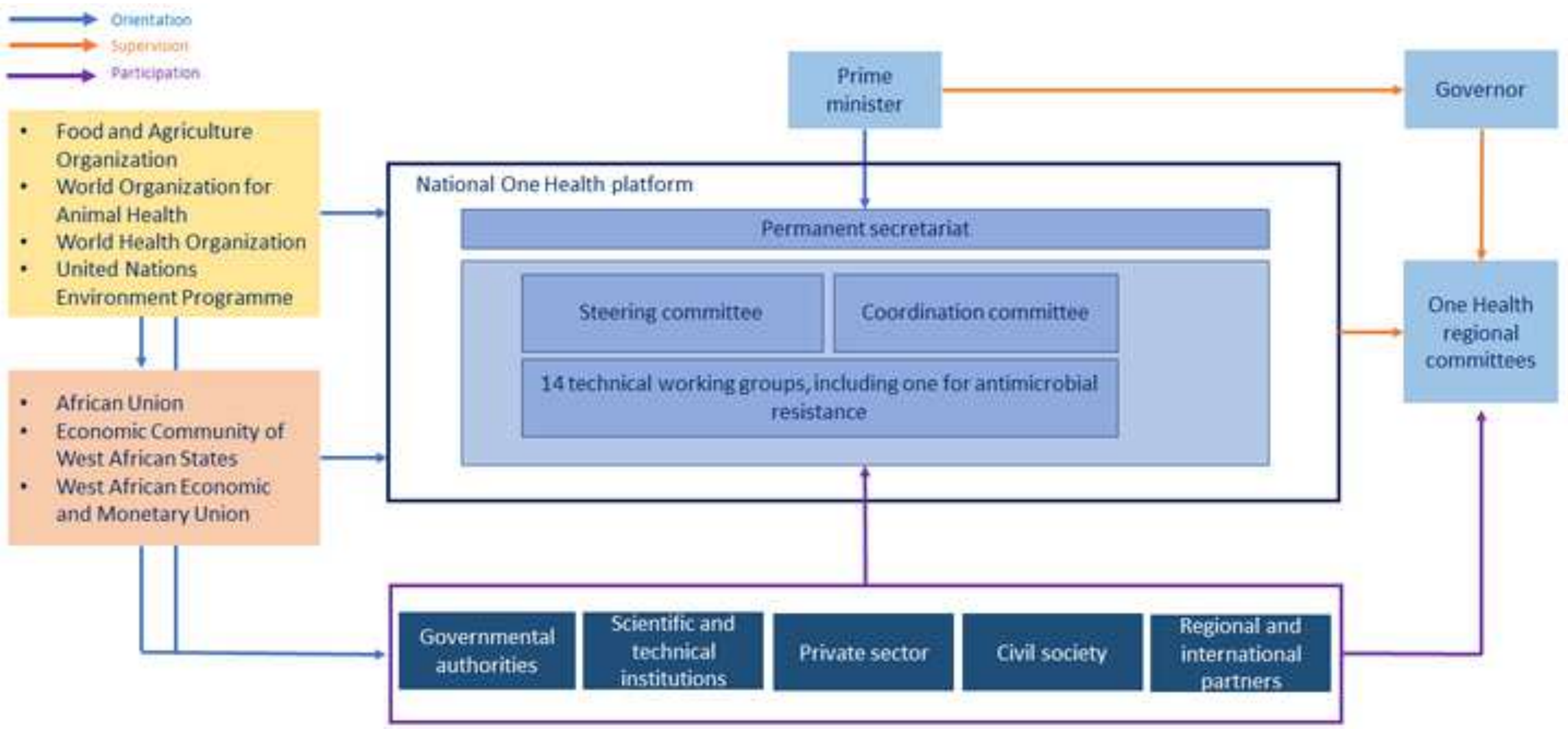

Supporting Information 1

Click here to access/download

Supporting Information

20250908_Supplementary file 1.docx

Supporting Information 2

Click here to access/download
Supporting Information
20250908_Supplementary file 2.docx
