# Supplementary material for: Governing antibiotic resistance through One Health: Insights from the political and legal landscape in Senegal
Source: PLOS Glob Public Health. 2026 Mar 12;6(3):e0005889. doi: 10.1371/journal.pgph.0005889 (PMC12981456; doi:10.1371/journal.pgph.0005889)
Supplement: Attachment — Submitted filename: Response_to_reviewers.pdf [file pgph.0005889.s004.pdf]

RESPONSE TO REVIEWERS

We sincerely thank the reviewers for their valuable time, thoughtful comments, and insightful suggestions on our manuscript. We appreciate the opportunity to revise our work and have carefully considered all the points raised. In response to this major revision, we have made substantial changes to the manuscript, as detailed below. We believe these revisions have significantly strengthened the clarity, relevance, and overall quality of the paper.
Below, we provide a point-by-point response to each comment. Reviewer comments are presented in *italics*, followed by our detailed responses and explanations of the changes made

Answers to reviewer #1's comments

1. Line 242: Is it AMR stewardship of an antimicrobial stewardship plan?

Thank you for spotting this mistake. Indeed, it is an AM stewardship plan and not an AMR stewardship plan. The manuscript has been corrected accordingly (see line 251 in the marked version of the revised manuscript)

2. The discussion section (461-492) is not clear. It does not speak to Senegal but to the African continent as a whole. The section should tie the findings in this paper to the continental ecosystem.

We have revised this section to build on the findings of this study and show how similar situations can be found in other countries in the region. See lines 470-477, and 485-489 of the marked version of the revised manuscript.

3. The same is noted from lines 493 - 551; it focuses more on the international ecosystem. The discussion should focus on what these findings mean and then flow into the recommendations

Thank you for your suggestion. We have revised the manuscript to put more emphasis on the findings of the study and how they are linked to the continental ecosystem (see lines 503-510). We have then made a clearer link to the next paragraph which includes our recommendations.

4. The recommendations are not clearly stated, and the conclusion still highlights the global level. Except this can be linked to the situation in Senegal, then it is not clear on how these recommendations impact the local situation

We have revised the manuscript to make our recommendations (see lines 588-602 in the marked version of the revised manuscript), and the conclusion clearer particularly in the Senegalese context. We have also emphasized that what this study has highlighted in Senegal

has also been highlighted in other African countries with limited resources, which allows us to extend the recommendations to this category of countries.

Answers to reviewer #2's comments

1. Would be helpful to have a structured abstract if possible.

Unfortunately, a structured abstract is not in line with the journal's requirements: "While the Abstract is conceptually divided into three sections (Background, Methodology/Principal Findings, and Conclusions/Significance), do not apply these distinct headings to the Abstract within the article file." However, the abstract follows the three sections, even with no explicit reference to them.

2. Would break the sentence in lines 66 - 69 into two sentences: "One Health is defined as an integrated, unifying approach that aims to sustainably balance and optimize the health of people, animals, and ecosystems. The One Health concept recognizes the links between and interdependence of the health of humans, domestic and wild animals, plants, and the wider environment (including ecosystems)."

We have broken the sentences into two sentences as suggested: "One Health is defined as an integrated, unifying approach that aims to sustainably balance and optimize the health of people, animals and ecosystems. The concept recognizes the links between and interdependence of the health of humans, domestic and wild animals, plants, and the wider environment (including ecosystems)". See lines 65-69 in the marked revised manuscript.

3. Would do the same with lines 70 - 75 as above. Currently the sentences is very long.

We understand your concern about this sentence, but it is only long because of the definitions of the acronyms of the institutions mentioned. We prefer to keep it as it is and let the 2 examples together in order not to make the text more cumbersome.

4. Line 81 should start with a new paragraph about what Senegal is doing.

We have introduced a line break to start a new paragraph with regard to Senegal. See line 80 in the marked revised manuscript.

5. With the methods, you begin with defining the concepts but I would start with your actual methodology (lines 107 to 112). This paragraph should stand alone and the definitions can come after.

Thank you for your suggestion. We have swapped the paragraphs, and put the one related to our methodology first. See lines 93-103 of the marked revised manuscript.

6. Lines 97 & 99 would suggest just calling it ABR instead of ABR issue.

We have replaced ABR issue by ABR as suggested, lines 113 and 11 in the marked revised manuscript.

7. *Line 101 would remove "and" and place a comma: "sometimes underpinned by an institutional and legal framework, and facilitated by effective management, transparent mechanisms, and relationships."*

Thank you for your suggestion. However, the term “transparent” refers to both mechanisms and relationships. We have therefore reworded the sentence to clarify this point: « For this study, we have adapted this definition to include the One Health dimension applied to the ABR issue; we referred to One Health governance of ABR as the process by which stakeholders collaborate and coordinate their collective action to develop and implement policies to manage ABR , sometimes underpinned by an institutional and legal framework, and facilitated by effective management, as well as transparent mechanism and relationships.” See line 116 of the marked revised manuscript.

8. Its seems like this is more of a grey lit review and not an academic lit review, I might specify this.

You are right and we have updated the manuscript to mention it. See line 130 of the marked version of the revised manuscript.

9. Was academic literature assessed at all? And if not, why?

We did not retrieve any specific literature describing in details politico-institutional framework for ABR management at regional and national levels with regard to the characteristics we were interested in. However, a few scientific articles address this topic and have been used to identify certain documents of interest included in this study and to fuel discussion of the results. We have clarified this point line 131-134 if the marked version of the revised manuscript.

10. Would recommend creating a flow diagram showing the number of experts invited, number who participated, number that conducted in-person interview, and number who did not. In this diagram I would also include expert counts with the sector and occupation the expert belongs to so readers can visualize this data.

Thank you for this recommendation. We have incorporated this information into the manuscript; however, we opted for a table, which we felt was a more appropriate structure for presenting all of this information. See lines 168 of the marked version of the revised manuscript.

11. For interviews not conducted in person, would add a line on how they were carried out (e.g. phone, virtual platform, written).

Thank you for your suggestion. We have clarified in the manuscript that interviews that were not conducted in person were conducted using a video conferencing platform. See lines 156-158 of the marked version of the revised manuscript.

12. Remove lines 159 - 163 as you have and ethics section and this is repeated.

Thank you for pointing out this repetition. We have removed this paragraph and move the ethic section at the beginning of the method section as requested by the journal.

13. The first header "The politico-institutional framework for antibiotic resistance management in Senegal" aligns with the first objective in the methodology but the subsequent headers don't. I would keep the headings consistent with your objectives and add subheadings as needed to improve clarity (i.e. second header in results: Drivers of One Health governance and ABR management, third header: Improving effective One Health governance).

We have revised the wording of the objectives (see lines 93-98 of the marked revised manuscript) and the section on results to ensure better alignment between the two. The first part of the results therefore responds to the first objective of the study, and the second part to the second objective of the study. In this second part of the results, as the areas for improvement described are specific to each capacity to address their limitations, we have chosen to leave them after the description of the situation with regard to that capacity for greater readability. However, we have changed the title of the second header to be more consistent with the content. We hope you are satisfied with this change. The new title is « The One Health governance capacities for antibiotic resistance in Senegal, limits and ways of improvement » See lines 297-298 of the marked version of the revised manuscript.

14. Line 329 sounds close to a claim made in a discussion section not the results. Would remove and start with the next line 'However, most programs..."

We agree that this sentence appears to be a claim without any verbatim evidence to support it. We have therefore removed it from the results. See lines 339-340 in the marked version of the revised manuscript.

15. Similar for line 349, Would recommend removing and presenting the finds in lines 352. If these statements are from your interviewees, then I would directly specify that and present it as such, again it reads like author comments which should be held for the discussion.

In this case, these are indeed results drawn from the analysis of the document database presented in the first part of the results. This allows us to contextualize the comments made by the respondents. However, we have reworded this section to make this connection and be more neutral: “The analysis of the legal framework shows that Senegal has a fairly

comprehensive regulatory framework for managing ABR”. See lines 362-363 of the marked version of the revised manuscript.

16. Similar for lines 377 - 385,

Again, this statement is coming from our literature review. To avoid confusion, we have mentioned this in the revised version of the manuscript (line 388) and made reference to the legal instruments where this information is available (line 392).

17. Similar for lines 394 - 397

This statement is derived from the thematic analysis of the actors' discourse. However, we recognize that the wording of the sentence does not necessarily allow for a connection to be made with the verbatim quotes used later to illustrate the statement. We have therefore reworded this section. See lines 405-407 in the revised marked manuscript.

18. 424 - 426 similarly to #2, read more like a discussion section or like a recommendation that would be brought forward in the discussion section rather then the results section. I would recommend beginning the section with the objective findings of your Key Stakeholder interviews.

These statements are derived directly from the literature review and participants' comments, as partially shown by the verbatim quotes used below to illustrate these results. We have added a verbatim quote to support our statement that stakeholders have gained experience in collaboration in recent years, particularly with the implementation of the One Health platform. See lines 417-421 in the revised marked manuscript.

19. Lines 377 - 385 need references unless all of these claims were from the interviews -if so this needs to be stated. Right now, line 388 discusses hospital laboratories but I can't find any interview excerpts that speak to health system strengthening from COVID or Ebola. So the lines read like the author is making these claims. If these are the authors claims, would suggest moving these down to the discussion and adding citations here.

See answer to comment 16 (as we understood it was the same suggestion).

21. There is a lot of material here but the discussion section doesn't explicitly state the key findings. This section very rarely engages with the data generated through the interviews and I can't see where it mentions the 5 key themes that were introduced in the results. The section reads largely as a theoretical or literature-based commentary. To strengthen it I would recommend explicitly interpreting each key finding of your results (here is where you could add some commentary that was in the results section), integrating what interviewees said, and highlighting the study’s contributions to AMR governance.

Thank you for your comment. We have deeply revised the manuscript to better anchor the discussion in the study's findings. As a result, the discussion is now structured as follows. We first built on the Senegalese case study to show that One Health governance of AMR in Senegal relies mainly on the One Health platform and action plan, but that this political and institutional environment is struggling to deliver the expected results. We took the opportunity to show that Senegal is not an isolated case and that these observations are also made in other resource-limited African countries. We then discuss how to improve the situation can be improved in Senegal, and by extension in other LMICs with similar contexts and aid regimes, by improving institutionalization at the global and national levels with its associated limitations.

22. I don't think you need subheadings in the discussion section and if keeping, I would again match them up to your original objectives.

As suggested, we have removed the subheadings in the revised version of the manuscript.

23. Line 489 needs a citation

We have revised this sentence and added a citation. See line 498-500 in the marked version of the revised manuscript.

24. Some language in this section should be more normative to maintain an analytical tone (e.g. "This calls into question the legitimacy..." "....rather than real strategic governance instruments"), all true but would try to keep more neutral language.

Thank you for your comment. We have revised the manuscript to keep a more analytical tone as suggested. See lines 500-502 in the marked version of the revised manuscript.

25. Line 545 is great, it speaks to the barriers countries face when tailoring their policies to the local context. I would build on that, reiterate what this study adds to the current literature, and what future work is needed beyond this study to continue to build and improve AMR governance within Senegal and other LMICs.

In the revised manuscript, we have placed greater emphasis on the contribution of the case study in Senegal on the difficulty of operationalizing national policies and have used these elements to develop a specific paragraph with recommendations for improving One Health governance in Senegal and in countries in similar situations. See lines 588-602 in the marked version of the revised manuscript.

26. Lines 548, I agree that chasing a score is not the goal but might a public score increase transparency, which is needed in AMR governance and policy? I might mention that here.

We have indeed pointed out that assessments are important in the policy cycle but that, in light of our findings, they may not be suitable for supporting the development of One Health policies because they do not explore all the important dimensions to be taken into account in One Health governance. We have included a recommendation to improve evaluation. See lines 600-602 in the marked version of the revised manuscript.

27. Need a citation and maybe example for this claim in line 549: "...sometimes to the detriment of other national priorities.."

We have added a reference to illustrate this claim (see line 586 in the marked version of the revised manuscript).

28. A limitation section is needed.

We added a paragraph on the limitations of this study and emphasized that the results of this study were only valid at the time it was conducted due to the highly evolving nature of One Health governance. See lines 604-620 of the marked version of the revised manuscript.

29. Would try to shorten this to one concise paragraph.

We have shorten the conclusion.

Answers to reviewer #3's comments

1. The first, is just to add some further clarifications around the methodology employed. As highlighted in the returned PDF this is regards to the number of interviews and interviewees, giving the reader a clearer indication of how many interviews were conducted in a group setting and subsequently some clearer outline of what languages the interviews were conducted in, and if not carried out in English, how and when the translation process took place (pre-transcription, pre-analysis, or just for the purpose of reporting quotes in the manuscript)?

We have provided details on the nature of the participants and how the data was collected and managed in Table 1 (line 166) and in the body of the manuscript. See lines 156-158 of the marked version of the revised manuscript.

2. The second, not outlined in the returned PDF, is that wherever possible/relevant in the Discussion section could you add more content on the main findings of the interviews. Better integrating the key themes from the interviews into the sections primarily focused on the policy analysis and implementation would further bolster the arguments made and make the connection between the two main methodological components of the research clearer for the reader

Thank you for your comment. We have deeply revised the manuscript to better anchor the discussion in the study's findings. As a result, the discussion is now structured as follows. We first built on the Senegalese case study to show that One Health governance of AMR in Senegal relies mainly on the One Health platform and action plan, but that this political and institutional environment is struggling to deliver the expected results. We took the opportunity to show that Senegal is not an isolated case and that these observations are also made in other resource-limited African countries. We then discuss how to improve the situation can be improved in Senegal, and by extension in other LMICs with similar contexts and aid regimes, by improving institutionalization at the global and national levels with its associated limitations.

In addition, we have clarified the connection between the two methodological components. See lines 98-103 in the marked version of the revised manuscript.

3. Could you just clarify where this discrepancy comes from? For example, were 11 people interviewed in a pair rather than 1 on 1?
If its not immediately easy to understand/describe - please add an endnote here to guide the reader. If some interviews were conducted in group settings rather than 1 on 1, then this needs detailing in the body-text as it does affect the comparability of the data gathered (albeit only in a small way). Line 148

We have provided details to clarify why there is a discrepancy between the number of interviews and the number of interviewees. In total, we interviewed 37 people across 26

interviews (21 individuals interviews and 5 collective interviews). See lines 162-163 of the marked version of the revised manuscript.

4. Could you add into this section a note about the language(s) that the interviews were conducted into, and whether they were analyzed in this language or translated prior to analysis. (Line 148)

We have updated the manuscript to clarify the study language. The whole the study was conducted in French (data collection, management, and analysis). See lines 165-166 of the marked version of the revised manuscript.

5. Could you reword this? As it is it gives too much agency to institutions, rather than the individuals you interviewed. (Line 197-198)

Thank you for your comment. However, we prefer to leave things as they are, because in the design of our study, one actor equals one institution. Even though we interviewed several people within an institution, we did not consider that we had collected as many points of view as there were people present, but rather the point of view of a single actor. It seems more accurate to say that we interviewed 26 actors rather than 37. We hope this explanation satisfies you.

6. Could you make the numbers in this second part a little clearer? If the numbers that follow 11 (17, 10, 3) are all standards and guidelines, how come they are more numerous in total? (Line 271)

We propose a rewording of this section to improve the clarity of the manuscript. See lines 277-283 of the marked version of the revised manuscript.

7. First time the acronym/abbreviation has been introduced, please write in full. (Line 281)

Thank you for your vigilance. We have defined the acronym in line 290 of the marked version of the revised manuscript.

8. Were any of these frustrations expressed by interviewees? If so, perhaps include an example here to further evidence and detail this frustration. (Line 582-583)

Since we do not have a verbatim excerpt to illustrate these remarks, that arise from our observation of the policy formulation in Senegal, we have put the sentence in the conditional tense. See lines 541-543 of the marked version of the revised manuscript.
